# Excisional Biopsy of the Pyogenic Granuloma in Very High-Risk Patient

**DOI:** 10.1155/2018/5180385

**Published:** 2018-10-14

**Authors:** Dirceu Tavares Formiga Nery, José Ranali, Darceny Zanetta Barbosa, Helvécio Marangon Júnior, Rafael Martins Afonso Pereira, Patrícia Cristine de Oliveira Afonso Pereira

**Affiliations:** ^1^School of Dentistry, Catholic University of Brasília (UCB), Brasília, DF, Brazil; ^2^Department of Pharmacology, Anesthesiology and Therapeutics, School of Dentistry, University of Campinas (UNICAMP), Piracicaba, SP, Brazil; ^3^Department of Oral and Maxillofacial Surgery and Traumatology, School of Dentistry, Federal University of Uberlandia (UFU), Uberlândia, MG, Brazil; ^4^School of Dentistry, University Center of Patos de Minas (UNIPAM), Patos de Minas, MG, Brazil

## Abstract

Oral surgery to remove pyogenic granuloma in a high-risk patient is reported. A 47-year-old man with gastroesophageal reflux disease, diabetes mellitus II, dyslipidemia, and chronic coronary insufficiency (myocardial infarction within 2 years) with episodes of unstable angina was submitted to an excisional biopsy of hemorrhagic lesion in the lingual right mandibular gingiva. During dental treatment, the arterial blood pressure, oxygen saturation, heart rate, and electrocardiogram were monitored. Local anesthesia was performed with 0.45 ml of 3% prilocaine with 0.03 IU/ml felypressin. The anticoagulant therapy was not interrupted. No local or systemic complications were noticed during or after the surgery.

## 1. Introduction

Coronary artery disease (CAD) is highly prevalent in industrialized countries, and although prevention, diagnosis, and medication have improved, it is still the main cause of death [1, 2]. When CAD is symptomatic, angina pectoris is the presenting sign. Unstable angina pectoris (UA) and myocardial infarction (MI) are more critical situations and demand prompt medical care [3].

Because of the high prevalence of CAD and the improved preventive measures leading to patients' teeth being preserved into advanced age, the general dental practitioner will more frequently encounter CAD [3, 4]. There are no contraindications to elective dental treatment of patients with stable angina [3]. UA and MI in the period of 6 months after their occurrence were designated by the American Society of Anesthesiologists (ASA) as class IV patients, limiting their dental care to urgency treatment [2, 5–7]. However, in view of the current technological advances in both peri-MI and post-MI treatment and assessment, these guidelines have been revised [5, 6].

If ASA IV patients (UA or IM) with dental pain are given no treatment, this may aggravate the ischemic attacks due to pain and anxiety, thus leading to increased secretion and release of catecholamines [2–7]. On the other hand, the dental treatment itself may endanger the patient's life, if it is not administered and monitored properly [2, 5, 6, 8].

This article reports on the safe management and monitoring of an ASA IV patient during a dental procedure: excisional biopsy of a pyogenic granuloma.

## 2. Clinical Presentation

**Figure 1:**
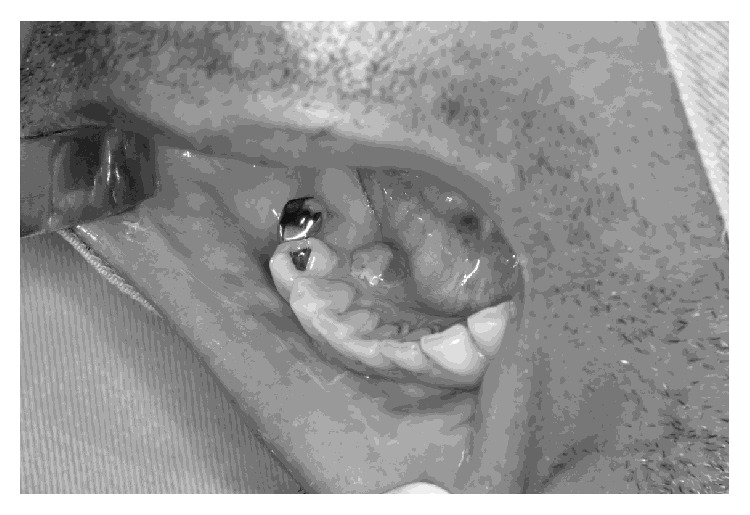


A 47-year-old man presenting with active CAD and considered to be a very high-risk patient (ASA IV) was referred to the Dentistry School of Uberlândia Federal University exhibiting a solitary nodule, 8 mm in diameter, in the lingual right mandibular gingiva (Figure 1), with spontaneous and intermittent bleeding.

Physical examination revealed a painless lesion, within a well-demarcated violet-colored (hemorrhagic) margin, friable in consistency, and of unknown origin, involving the lingual gingiva in the area of teeth # 43-44. According to the patient, the lesion had been present for approximately two months, exhibiting slow growth. Radiographic examination showed no teeth and/or bone involved. The initial diagnosis was pyogenic granuloma.

**Figure 2:**
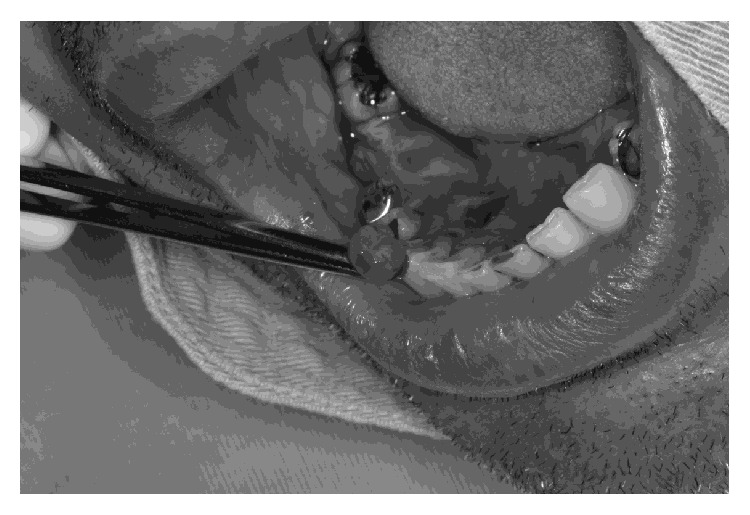


The patient's medical history showed gastroesophageal reflux disease, diabetes mellitus II, dyslipidemia, and chronic coronary insufficiency (myocardial infarction within 2 years) with episodes of unstable angina. The patient had been submitted to myocardial revascularization surgery (2 years previously); first, right coronary artery angioplasty (CAA) with stent (within 1 1/2 year); second, CAA (within 1 year), due to intrastent restenosis; third, CAA (about 9 months before) with new restenosis and stent implantation in ACD and previous descending artery (ADA); fourth, stent implantation due to restenosis (within 3 months); and finally, 2 months ago, this patient presented second degree atrium-ventricular block (mobitz II), being submitted to definitive artificial cardiac pacemaker implantation. The patient was receiving anticoagulants, oral nitrates, beta blockers, and calcium channel antagonist. The treatment of choice was an excisional biopsy with curettage (Figure 2), due to the possibility of recurrence. During dental treatment, blood pressure, heart rate, continuous electrocardiogram (ECG), and oxygen saturation were monitored. The emergency service was on stand-by at the ambulatory, and it could be brought into action if necessary. The patient's physician was consulted, and none of the medication being administered to the patient was suspended, including acetylsalicylic and ticlopidine hydrochloride. The patient's international normalized ratio (INR) on the day of the surgery was 2.8.

The surgical appointment started at 9:30 am. No sedation method was used, as the patient was not anxious and the procedure would be performed in a short time. Preoperative vital signs were as follows: blood pressure (BP) 110/86 mmHg, heart rate (HR) 57 bpm, and 96% oxygen saturation (OS). On that day, the patient complained of chest pain.

**Figure 3:**
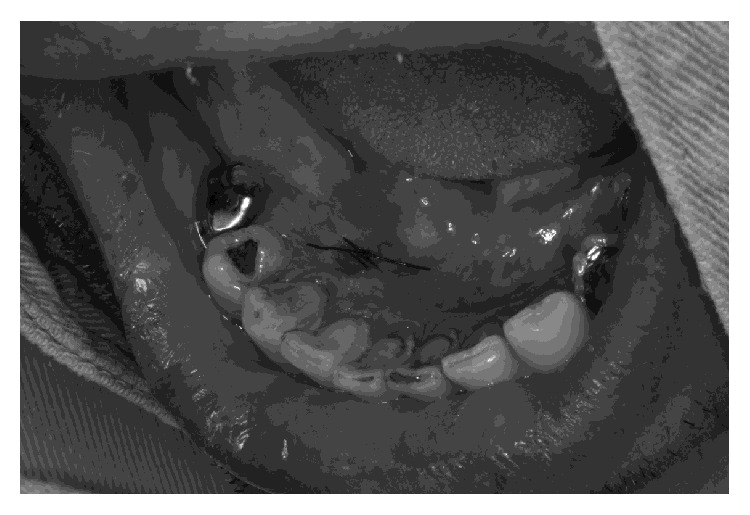


After local anesthesia with 0.45 ml of 3% prilocaine with 0.03 IU/ml felypressin, the nodule was excised and sent for histopathologic evaluation. No active bleeding was observed during or after surgery (Figure 3). During dental treatment, the patient did not complain of exacerbation of the symptoms, such as chest pain and dyspnea, or show marked hemodynamic change that necessitated discontinuation or postponement of the surgery, which was completed in 30 minutes. After treatment, the patient was carefully observed for vital signs, cardiac symptoms, pain, and ECG irregularities for 24 hours. Postoperative vital signs were as follows: BP, 111/90 mmHg; HR, 56 bpm; and OS, 95%. Acetaminophen (750 mg each 6 h, for 24 h) was prescribed to control postoperative pain.

**Figure 4:**
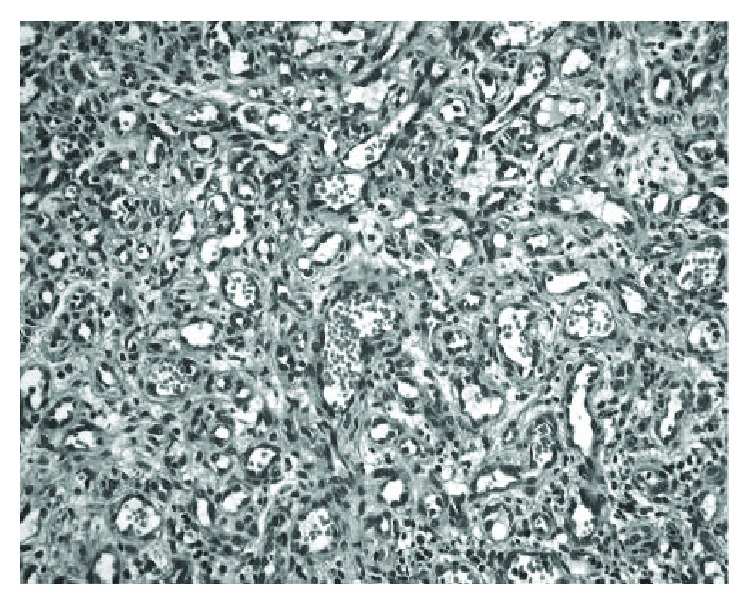


Histopathologic examination confirmed the previous diagnosis of pyogenic granuloma (Figure 4). Follow-up examinations have been made every 30 days, to rule out recurrence of the lesion. No local or systemic complications were observed in a period of one month.

## 3. Discussion

The advances in prevention, diagnosis, and treatment of cardiovascular diseases have led to an increased survival of patients, although this group of diseases continues to be the first cause of death in industrialized countries. For successful dental management of these patients, as with any other disease, a complete medical history and physical examination, including blood pressure, heart rate, and respiratory function, are indispensable. When indicated, medical consultation must be sought [9–11].

Many authors have recommended elective dental treatment only after 6 months of myocardial infarction and with stable angina [7, 9, 12–16]. Therefore, an oral surgery should be postponed in patients with arterial coronary disease, due to the high risk of reinfarction and cardiac arrest [7]. Alexander et al. [17], on the contrary, state that patients with coronary artery disease have a 1.1% perioperative rate of MI after noncardiac surgery, compared with a rate of 0 to 0.7% in the general population.

According to Niwa et al. [6], dental treatment is possible in high-risk patients, but should be limited to procedures of short duration, with good pain control during and after the procedure. In their study, 63 patients received dental treatment after complaints of toothache or masticatory disorder during hospitalization, under local anesthesia with 3% prilocaine with 0.03 IU/ml felypressin, in a maximum period of 30 minutes. The procedures would be stopped if cardiovascular conditions showed signs of deterioration. In some of the cases, sedation (nitrous oxide or diazepam IV) was used. All the procedures were carried out without interruption, and 8 patients had angina in the period of one week after the procedure, 5 of them related to the dental treatment. Of importance is the fact that the incidence of postoperative complications was higher in patients with history of chest pain 2 weeks before the dental treatment and in those who failed to clear the Master Test Single stress test.

In the case presented here, although the patient had a history of unstable angina with complaints of chest pain daily, the procedure was accomplished without postoperative complications. The 24 h ECG monitoring showed no postoperative complications without any new ST depression. Although it is recommended in the literature that patients with cardiovascular disease should be treated under sedation, it is interesting that in the Niwa et al.'s study and in the present case, no relation was observed between the presence of complication and whether or not pharmacological sedation was used. Iatrosedation and the patient's confidence in the dentist are of great importance. In the present case, the medical care is a factor that could also have increased the patient's confidence.

According to Findler et al. [2] and Chapman [7], a prophylactic dose of nitrates a few minutes before the appointment begins is recommended for patients with history of angina. However, this patient presented with continuous chest pain and used isosorbide dinitrate every 4 hours. Due to the risk of hypotension with reflex tachycardia [7], this recommendation was not followed.

As the clinical diagnosis was a pyogenic granuloma, the treatment followed the established protocol, consisting of surgical excision and removal of etiological factors [18–20].

According to Campbell et al. [21], patients can safely undergo routine outpatient oral surgical procedures without alteration of their regular therapeutic anticoagulation regimens [21–26]. It is necessary to establish the prothrombin time/INR on the day of the dental procedure or dental surgery, to ensure that it is in a safe or therapeutic range [23, 27]. Serum level is monitored via the corrected prothrombin time (international normalized ratio—INR). The optimal INR for most conditions is up to 3.018. The case presented here supports this statement, as no excessive bleeding was observed during or after the surgery, with an INR of 2.8 and no anticoagulant withdrawal.

It should be emphasized that in spite of the recommendation that cardiovascular patients should only be treated at least 60 days after myocardial infarction and with clearing the exercise stress testing [5, 6], there are cases such as presence of pain or lesions that must be removed and diagnosis that demands treatment under far from ideal conditions, as in the present case. This shows that it is possible, even in such cases, to offer patients safe treatment.

Dental treatment must therefore be carefully conducted, with proper pain and anxiety management to minimize any elevation in endogenous catecholamine [2, 3, 5, 6] which could cause a number of undesirable effects, such as increased blood pressure and alterations in heart rate.

Therefore, the local anesthetic solution and technique must be those that offer appropriate efficacy and duration without the need for another injection during the procedure to avoid unnecessary pain and stress. In the present case, prilocaine with felypressin was the choice [6], and acetaminophen was prescribed for avoiding postoperative pain.

To avoid additional risks and offer safe treatment in high-risk patients, as in the present case, oral surgery must be performed with cardiovascular monitoring in a medical facility with emergency support.

## References

[1] Kammel W. B., Tham T. Y., Hurst J. W. (1990). Incidence, prevalence and mortality of cardiovascular disease. *The Heart*.

[2] Findler M., Galili D., Meidan Z., Yakirevitch V., Garfunkel A. A. (1993). Dental treatment in very high risk patients with active ischemic heart disease. *Oral Surgery, Oral Medicine, and Oral Pathology*.

[3] Blanchaert R. H. (1999). Ischemic heart disease. *Oral Surgery, Oral Medicine, Oral Pathology, Oral Radiology, and Endodontics*.

[4] Jowett N., Cabot L. (2000). Patients with cardiac disease: considerations for the dental practitioner. *British Dental Journal*.

[5] Roberts H. W., Mitnitsky E. F. (2001). Cardiac risk stratification for postmyocardial infarction dental patients. *Oral Surgery, Oral Medicine, Oral Pathology, Oral Radiology, and Endodontics*.

[6] Niwa H., Sato Y., Matsuura H. (2000). Safety of dental treatment in patients with previously diagnosed acute myocardial infarction or unstable angina pectoris. *Oral Surgery, Oral Medicine, Oral Pathology, Oral Radiology, and Endodontology*.

[7] Chapman P. J. (2002). Chest pain in the dental surgery: a brief review and practical points in diagnosis and management. *Australian Dental Journal*.

[8] Sugimura M., Hirota Y., Shibutani T. (1995). An echocardiographic study of interactions between pindolol and epinephrine contained in a local anesthetic solution. *Anesthesia Progress*.

[9] Research, Science and Therapy Committee, American Academy of Periodontology (2002). Periodontal management of patients with cardiovascular diseases. *Journal of Periodontology*.

[10] Maloney W. J., Weinberg M. A. (2008). Implementation of the American Society of Anesthesiologists Physical Status classification system in periodontal practice. *Journal of Periodontology*.

[11] Herman W. W., Konzelman J. L., Prisant L. M. (2004). New national guidelines on hypertension: a summary for dentistry. *The Journal of the American Dental Association*.

[12] Goulet J.-P., Pe´russe R.´n., Turcotte J.-Y. (1992). Contraindications to vasoconstrictors in dentistry: part III: pharmacologic interactions. *Oral Surgery, Oral Medicine, Oral Pathology*.

[13] Pe´russe R.´n., Goulet J. P., Turcotte J. Y. (1992). Contraindications to vasoconstrictors in dentistry: part I: cardiovascular diseases. *Oral Surgery, Oral Medicine, Oral Pathology*.

[14] Silvestre F. J., Miralles-Jorda L., Tamarit C., Gascon R. (2002). Dental management of the patient with ischemic heart disease: an update. *Medicina Oral*.

[15] Rose L. F., Mealey B., Minsk L., Cohen D. W. (2002). Oral care for patients with cardiovascular disease and stroke. *The Journal of the American Dental Association*.

[16] Hupp J. R. (2006). Ischemic heart disease: dental management considerations. *Dental Clinics of North America*.

[17] Alexander R. W., Schlant R. C., Fuster V. (1998). *Hurst’s the Heart*.

[18] Regezi J. A., Sciubba J. J., Jordan R. C. K. (2017). *Patologia bucal: correlações clínicopatológicas*.

[19] Al-Khateeb T., Ababneh K. (2003). Oral pyogenic granuloma in Jordanians: a retrospective analysis of 108 cases. *Journal of Oral and Maxillofacial Surgery*.

[20] Bakshi J., Virk R. S., Verma M. (2009). Pyogenic granuloma of the hard palate: a case report and review of the literature. *Ear, Nose, & Throat Journal*.

[21] Campbell J. H., Alvarado F., Murray R. A. (2000). Anticoagulation and minor oral surgery: should the anticoagulation regimen be altered?. *Journal of Oral and Maxillofacial Surgery*.

[22] Campbell R. L., Langston W. G. (1995). A comparison of cardiac rate-pressure product and pressure-rate quotient in healthy and medically compromised patients. *Oral Surgery, Oral Medicine, Oral Pathology, Oral Radiology, and Endodontology*.

[23] Alexander R., Ferretti A. C., Sorensen J. R. (2002). Stop the nonsense not the anticoagulants: a matter of life and death. *The New York State Dental Journal*.

[24] van Diermen D. E., Hoogstraten J., van der Waal I. (2008). Dental procedures for patients using oral anticoagulation: new insights. *Nederlands Tijdschrift voor Tandheelkunde*.

[25] Caliskan M., Tukel H. C., Benlidayi E., Deniz A. (2017). Is it necessary to alter anticoagulation therapy for tooth extraction in patients taking direct oral anticoagulants?. *Medicina Oral Patología Oral y Cirugia Bucal*.

[26] Yagyuu T., Kawakami M., Ueyama Y. (2017). Risks of postextraction bleeding after receiving direct oral anticoagulants or warfarin: a retrospective cohort study. *BMJ Open*.

[27] Badoual T., Lellouche N., Bourraindeloup M. (2003). Pathology and dental care in the context of cardiovascular conditions: myths beliefs and realities. *Archives des maladies du coeur et des vaisseaux*.

